# Eating habits and obesity among Lebanese university students

**DOI:** 10.1186/1475-2891-7-32

**Published:** 2008-10-30

**Authors:** Najat Yahia, Alice Achkar, Abbass Abdallah, Sandra Rizk

**Affiliations:** 1 Department of Human Environmental Studies, Central Michigan University, USA; 2 Natural Science Division, Lebanese American University, Beirut, Lebanon

## Abstract

**Background:**

In the past year Lebanon has been experiencing a nutritional transition in food choices from the typical Mediterranean diet to the fast food pattern. As a consequence, the dietary habits of young adults have been affected; thus, overweight and obesity are increasingly being observed among the young. The purpose of this study is to assess the prevalence of overweight and obesity on a sample of students from the Lebanese American University (in Beirut) and to examine their eating habits.

**Methods:**

A cross-sectional survey of 220 students (43.6% male and 56.4% female), aged 20 ± 1.9 years, were chosen randomly from the Lebanese American University (LAU) campus during the fall 2006 semester. Students were asked to fill out a self-reported questionnaire that included questions on their eating, drinking and smoking habits. Also, their weight, height, percentage body fat and body mass index were measured. Body mass index (BMI) was used to assess students' weight status. Statistical analyses were performed using the Statistical Package for Social Sciences software (version 13.0) to determine overweight and obesity among students and to categorize eating habits.

**Results:**

This study showed that the majority of the students (64.7%) were of normal weight (49% male students compared to 76.8% female students). The prevalence of overweight and obesity was more common among male students compared to females (37.5% and 12.5% vs. 13.6% and 3.2%, respectively). In contrast, 6.4% female students were underweight as compared to 1% males. Eating habits of the students showed that the majority (61.4%) reported taking meals regularly. Female students showed healthier eating habits compared to male students in terms of daily breakfast intake and meal frequency. 53.3% of female students reported eating breakfast daily or three to four times per week compared to 52.1% of male students. There was a significant gender difference in the frequency of meal intake (P = 0.001). Intake of colored vegetables and fruits was common among students. A total of 30.5% reported daily intake of colored vegetables with no gender differences (31.5% females vs. 29.2% males). Alcohol intake and smoking were not common among students.

**Conclusion:**

In spite of the overall low prevalence of overweight and obesity in the studied sample, results indicate that university students would possibly benefit from a nutrition and health promotion program to reduce the tendency of overweight and obesity, especially among male students, and to improve students' eating habits.

## Background

Lebanon has been experiencing a nutritional transition in food choices during the past years from the typical Mediterranean diet into the fast food pattern [1]. Dietary habits of young adults are affected by the fast-food market. As a consequence, overweight and obesity are increasingly observed among the young. Obesity in combination with unhealthy life style, such as smoking and physical inactivity, may increase the risk of chronic diseases. In this regard, nutritional knowledge may act as a deterrent against fast-food trend. Thus, universities may contribute significantly in reducing the prevalence of obesity among the young population through the promotion of healthy eating habits. Universities may provide an ideal forum for reaching out to a large number of young adults through nutrition education programs that may positively influence students' eating habits by advocating for the adoption of healthy food choices. The purpose of this study was to assess the prevalence of overweight and obesity in a sample of students from the Lebanese American University and examine their eating habits. Assessing students' weight status and eating habits will help health educators to develop proper nutrition-related education programs that promote healthy food choices and good eating habits.

## Methods

### Design and sample

The study design was a cross-sectional survey conducted at the Lebanese American University (LAU) campus during the fall 2006 semester. A sample of 220 students (43.6% male and 56.4% female), aged 20 ± 1.9 years participated in this study. Students were recruited randomly by a trained student accompanied by an LAU professor. The response rate among students was high. Students who agreed to participate in this study were asked to sign a consent form according to Helsinki declaration.

### Data Collection

Data collection took place in two steps. The first step was to fill out the questionnaire and the second step was to perform the anthropometric measurements. Recruited students were asked to fill out a questionnaire related to their eating, drinking and smoking habits. The questionnaire was adopted from a previously published study where authors have standardized its use among university students [2]. Prior to questionnaire administration, students were informed by an LAU professor about the study. They were given instructions on how to fill out the questionnaire completely and truthfully. After filling out the questionnaire, anthropometric measurements, such as weight and height, percentage body fat and body mass index, were done. Weight, percentage body fat and body mass index measurements were determined using Tanita scale body fat analyzer 300A (BF 300, courtesy of Abbott Diagnostic Company, Lebanon). As fluctuations in body hydration status may affect body composition results, conditions for Tanita scale measurements were taken in the morning (at least three hours after waking up) when students were on an empty bladder, not having exercise, food or drink for at least three hours before having the measurements. Students were asked to wipe off the bottom of their feet before stepping onto the measuring platform, since unclean foot pads may interfere with conductivity. Height measurements were taken with a secured metal ruler. Students were asked to take off their shoes for height measurements. Body mass index (BMI) was used to assess students' weight status. According to guidelines stated by the National Institutes of Health, weight status was classified into four categories: underweight (BMI ≤ 18.5), normal weight (BMI between 18.5 – 24.9), overweight (BMI between 25–29.9), and obese (BMI ≥ 30) [3]. Normal range for percentage body fat was considered as follow: 10–20% for males and 20–30% for females.

### Data Analysis

Statistical analyses were performed using the Statistical Package for Social Sciences (version 13.0, SPSS, Inc) software. Analysis of variance (ANOVA) was used to examine differences in the anthropometric characteristics of students. Results were expressed as means ± SD (standard deviation). Parametric variables were analyzed using students' *t-test*, while chi-squared analyses were conducted for non-parametric variables. All reported *P *values were made on the basis of 2-sided tests and compared to a significance level of 5%; differences were considered statistically significant at P < 0.05.

## Results

### Characteristics of the students' sample and BMI values

Characteristics of the participated students are presented in Table 1. A total of 220 students (96 males and 124 females), with a mean age of 20 ± 1.9 years, participated in this study. The average weight and height of the participated students were 67.7± 15.8 kg and 168.0 ± 10.0 cm, respectively. Mean BMI and percentage body fat were 23.6 ± 4.1 and 23.7 ± 8.2, respectively.

**Table 1:** Characteristics of the participants (means ± SD)

*Variable*	*Total*	*Males*	*Females*
*Number of Students*	*N = 220*	*N = 96*	*N = 124*
*Age (years)*	*20 *± 1.9	*20 *± 2.0	20 ± 1.8
*Weight (kg)*	67.7 ± 15.8	79.6 ± 12.8	58.6 ± 11.2
*Height (cm)*	168.0 ± 10.0	177.0 ± 10.0	162.0 ± 10.0
*BMI*	23.6 ± 4.1	25.3 ± 3.7	22.2 ± 3.9
*% Body Fat*	23.7 ± 8.2	17.8 ± 4.5	28.3 ± 7.4

### Students' weight status based on BMI categories and percentage body fat

The outcome of this study indicated that the majority of the students (64.7%) were of normal weight (49% of the male students compared to 76.8% of the female students) as indicated in Table 2. Based on BMI classification, the prevalence of overweight and obesity was more common among male students compared to females (37.5% and 12.5% vs. 13.6% and 3.2%, respectively). In contrast, 6.4% female students were underweight as compared to 1.0% males. Students of normal weight had at the same time normal percentage of body fat (14.4% males vs. 26.7% females). Similarly, the obese male students had at the same time higher values of percentage body fat (24.4%) while the overweight male students had a percentage body fat that was slightly higher than the normal range (20.1%) (Table 3). In the underweight category, female students also had a lower percentage of body fat (16.9%) (Table 3).

**Table 2:** Prevalence of obesity among students based on BMI by gender

	Males		Females		Total	
Weight Status	*N=*	*Percentage*	*N=*	*Percentage*	*N=*	*Percentage*

Underweight*	1	1.0	8	6.4	9	4.1
Normal**	47	49.0	96	76.8	143	64.7
Overweight***	36	37.5	17	13.6	53	24.0
Obese****	12	12.5	4	3.2	16	7.2

Underweight (BMI ≤ 18.5), ** Normal (BMI between 18.5 – 24.9), *** Overweight (BMI between 25–29.9), **** Obese (BMI ≥ 30).

**Table 3:** Students' percentage body fat by gender

	Males		Females		*P *value
Weight Status	*N=*	Mean ± SD	*N=*	Mean ± SD	

*Underweight*	1	12.00	8	16.9 ± 2.11	0.0000
Normal**	47	14.4 ± 3.13	96	26.7 ± 4.75	0.0000
Overweight***	36	20.1 ± 1.94	17	39.0 ± 2.25	0.0000
Obese****	12	24.4 ± 2.29	4	42.4 ± 1.91	0.0000

* Underweight (BMI ≤ 18.5), ** Normal (BMI between 18.5 – 24.9), *** Overweight (BMI between 25–29.9), **** Obese (BMI ≥ 30).

### Students' eating habits

Eating habits of the students were compared by gender (Table 4). The majority (61.4%) reported taking meals regularly. Female students showed healthier eating habits compared to male students in terms of breakfast intake and meal frequency. 53.3% female students reported eating breakfast daily or three to four times per week compared to 52.1% male students. There was a significant gender difference in the frequency of meal intake (P = 0.001). The majority of students (52.7%) reported eating two meals per day. Among females, 56.5% reported eating two meals per day as compared to 47.9% males. Intake of colored vegetables and fruits was common among students. A total of 30.5% of the students reported daily intake of colored vegetables with no gender differences (31.5% females vs. 29.2% males). 27.3% of the students reported daily intake of fruits. Male students tend to eat more fruits daily as compared to females (29.2% vs. 25.8% respectively). Alcohol intake was not common among students. 25.3% of the studied students did not consume alcohol at all and the majority (57%) of students reported drinking alcohol rarely whereas 17.2% reported two/three times per week. The unhealthy eating practice was indicated by the fact that the majority (57.3%) of the students reported eating fried food more than three times per week. Among females, 54% reported eating fried food daily or three to four times per week compared to 61.4% males. Daily intake of snacks apart from regular meals was more common among females than males (55.6% vs. 50% respectively). Eating daily with friends and family was common among students (42.7%) with no differences in gender. Smoking was not common among students. 62.4% of the students reported that they do not smoke, 7.2% were ex-smokers and 30.3% were current smokers.

**Table 4:** Student's response to questions related to their lifestyle practices including eating habits, meal patterns, fruits and vegetables intake, fried food, alcohol consumptions and smoking habit.

			*Males*		*Females*		
*Questions*	*Levels*	*Total N=*	*N=*	*%*	*N=*	*%*	*P Value*

Do you take your meals regularly?	Always regular	135	62	64.6	73	58.9	> 0.05
	Irregular	85	34	35.4	51	41.1	

Do you take breakfast?	Daily	70	31	32.3	39	31.5	> 0.05
	Three or four times per week	46	19	19.8	27	21.8	
	Once or twice per week	37	18				
	rarely	67	28	29.2	39	31.5	

How many times do you eat meals except snacks?	One time	17	8	8.3	19	15.3	= 0.001
	Two times	116	46	47.9	70	56.5	
	Three times	55	24	25	31	25	
	Four times	22	18	18.8	4	3.2	

How often do you take snacks apart from regular meals?	Daily	117	48	50	69	55.6	> 0.05
	Three or four times per week	45	23	24	22	17.7	
	Once or twice per week	35	13	13.5	22	17.7	
	rarely	23	12	12.5	11	8.9	

How often do you eat green, red or yellow colored vegetables?	Daily	67	28	29.2	39	31.5	> 0.05
	Three or four times per week	68	26	27.1	42	33.9	
	Once or twice per week	55	26	27.1	29	23.4	
	rarely	30	16	16.7	14	11.3	

How often do you eat fruits?	Daily	60	28	29.2	32	25.8	> 0.05
	Three or four times per week	48	19	19.8	29	23.4	
	Once or twice per week	60	27				
	rarely	52	22	22.9	30	24.2	

How often do you eat fried food?	Daily	42	20	20.8	22	17.7	> 0.05
	Three or four times per week	84	39	40.6	45	36.3	
	Once or twice per week	60	24	25	36	29	
	rarely	34	13	13.5	21	16.9	

How often do you eat with friends and family?	Daily	94	41	42.7	53	42.7	> 0.05
	Three or four times per week	75	33	34.4	42	33.9	
	Once or twice per week	46	18	18.8	28	22.6	
	rarely	5	4	4.2	1	0.8	

What type of food do you think you should eat to have a balanced nutrition?	Mainly meat	19	9	9.4	10	8.1	> 0.05
	Mainly vegetables	25	8	8.3	17	13.7	
	Meat, vegetables and other variety of foods	164	74	77.1	90	72.6	
	others	12	5	5.2	7	5.6	

How often do you drink alcohol?	Two or three times per week	39	21	21.9	18	14.5	> 0.05
	Never	56	25	26	31	25	
	Rarely	125	50	52.1	75	60.5	

Please state your smoking history	Current smoker	67 (30.3%)	33	34.4	34	27.2	> 0.05
	Ex-smoker	16 (7.2%)	9	9.4	7	5.6	
	Never smoke	138 (62.4%)	54	56.3	84	67.2	

## Discussion

The purpose of this study was to assess the prevalence of overweight and obesity and examine eating habits in a sample of Lebanese University students. Body mass index was used to assess weight status. Based on BMI classification of weight status, findings of this study indicate that the majority of students were of normal weight. Normal weight was more prevalent among females (76.8%) as compared to males (49%), whereas, overweight and obesity were more common among male than female students. Students in the normal weight category had at the same time normal values of percentage body fat. Prevalence of overweight was 37.5% in males as compared to 13.6% in females. Obesity was more common among male students than females in the studied population. A total of 12.5% of the males were obese compared to 3.2% of the females. Moreover, obese students had at the same time higher values of percentage body fat. The lower rate of obesity among female students is expected since females are more cautious about their weight status than males, due to society perceptions which encourage females to be slender. This assumption was supported by the fact that only 1% of males were underweight as compared to 6.4% of females in this studied sample. Obviously, pictures of movie stars and models in fashion magazines and mass media have a strong impact on girls' body shape and image perception [4]. University girls see the shape and weight of fashion models as the ideal body shape and figure to attain. Girls with such strong body weight perception can be at risk of developing eating disorders [5]. Similar findings of prevalence of obesity among male university students were reported in recent studies [6,7]. In a study conducted among 749 students (68% females and 32% males) recruited from the State University of the Basque Country, prevalence rate of overweight and obesity was 25% in males compared to 13.9% in females [6]. Another study conducted among 989 medical students (527 men, 462 women) from the University of Crete reported that approximately 40% male students and 23% female students had BMI > 25 kg/m^2 ^[7]. High prevalence rate of overweight and obesity was also reported in a study conducted in Kuwait University among 842 students [8], at 32% and 8.9%, respectively. In the United Arab Emirates, a cross-sectional survey conducted among 300 male students reported that the prevalence rate of obesity was 35.7% in males and this figure was higher than the rate in females [9]. In terms of eating habits, university students usually do not follow healthy eating habits. The typical university student diet is high in fat and low in fruits and vegetables [10]. Students often select fast food due to its palatability, availability and convenience. A previous survey by the American Dietetic Association indicated that obesity, or being severely overweight, is a fast-food related issue [11]. The Healthy people 2010 objectives include a focus on nutrition and obesity prevention [12]. In this study, data analyses of students' eating habits revealed that the majority of students eat meals regularly and eat breakfast daily or three to four times per week. 52.7% of the students eat meals two times per day. However, there was a significant gender difference in the frequency of meal intake in the studied sample (*P *= 0.001). As expected, intake of colored vegetables and fruits was also common among students. Alcohol intake and smoking were not common among students. The majority of students believe that eating meat, vegetables and other foods will provide them with a balanced diet. 77% male students and 73% female students in this study agreed that it is important to eat a variety of foods to have a balanced and nutritious diet. A study conducted at Midwestern University among 105 male and 181 female students, reported that 94.4% of the students agreed that it is important to eat a variety of foods for good health [13]. In another study, healthful diet was classified as a diet that included more fruits and vegetables, and less fat [14]. Daily intake of snacks was reported by the majority of students. The unhealthy eating habit of students was noticed in the intake of fried food (majority reported eating fried food three or four times per week). Frequent snacking and eating fried food can adversely affect students' health status, given the abundance of energy dense and high fat ingredients they contain. Improving students' knowledge about nutrition and healthy eating habits may promote healthy body weight management among students and reduce the prevalence of overweight and obesity. A recent study conducted among college students reported that increased knowledge of dietary guidance, *Dietary Guidelines for Americans 2005*, appeared to be positively related to more healthy eating patterns thus the better eaters had a higher level of knowledge about nutrition[15]. Therefore, developing nutrition education programs that promote healthy eating habits for university students should be encouraged. Alcohol intake and smoking were not common in our sample of students. National data on alcohol intake and the prevalence of smoking among university students in Lebanon are limited. A previous study conducted among 1850 Lebanese university students reported that the prevalence of drinking alcohol was found to have increased through the 1990s. However, the author stated that protective factors, such as belief in God (irrespective of the students' religion), practice of faith, and family or peer negative attitudes towards excessive drinking, were associated with less frequent experimentation with alcohol [16]. A previous study conducted among 2443 students from 13 public and private schools in Greater Beirut reported that the prevalence rate of cigarette smoking was 2.5% [17]. In a recent study, namely the Lebanon Global Youth Tobacco Survey (GYTS), conducted among 5035 students aged 13–15 years from 50 schools reported that the prevalence rate of students who were current cigarette smokers was 8.6% and 33.9% were current water-pipe smokers. The GYTS indicated that half of students who were current smokers expressed their desire to stop smoking [18].

## Limitations

The findings of this study are limited by the use of a sample of students from just one university which may not be a representative of all university students in Lebanon. Furthermore, students attending the Lebanese American University are usually of high socio-economic standards; therefore, samples from different universities may provide a more inclusive picture of university students taking into consideration religion and socio-economic status. However, baseline information about weight status and eating habits among a sample of university students was certainly obtained from the present study.

## Conclusion

Despite the low prevalence of overweight and obesity in the studied university students' sample, results indicate that university students would benefit from a nutrition and health promotion program to reduce the tendency of overweight and obesity among students, particularly males, and to improve students' eating habits.

## Competing interests

The authors declare that they have no competing interests.

## Authors' contributions

NY carried out questionnaire design, manuscript preparation and total coordination of the study. AA contributed to data analysis. AA contributed in the data collection and data entry. SR was actively involved in study's implementation and total coordination of study data collection and analysis. All authors read and approved the final manuscript. 

## Sources of Funds

No external funds. Authors funded this study.
